# Exploiting gene dependency to inform drug development for multiple myeloma

**DOI:** 10.1038/s41598-022-16940-7

**Published:** 2022-07-26

**Authors:** Molly Went, Phuc H. Hoang, Philip J. Law, Martin F. Kaiser, Richard S. Houlston

**Affiliations:** 1 Division of Genetics and Epidemiology, The Institute of Cancer Research, London, SW7 3RP UK; 2 Present Address: Division of Cancer Epidemiology and Genetics, National Cancer Institute, Bethesda, MD USA

**Keywords:** Cancer genetics, Myeloma

## Abstract

Despite recent advances in therapy, multiple myeloma essentially remains an incurable malignancy. Targeting tumour-specific essential genes, which constitute a druggable dependency, potentially offers a strategy for developing new therapeutic agents to treat MM and overcome drug resistance. To explore this possibility, we analysed DepMap project data identifying 23 MM essential genes and examined the relationship between their expression and patient outcome in three independent series totalling 1503 cases. The expression of *TCF3* and *FLVCR1* were both significantly associated with progression-free survival. *IKBKB* is already a drug target in other diseases, offering the prospect of repurposing to treat MM, while *PIM2* is currently being investigated as a treatment for the disease. Our analysis supports the rationale of using large-scale genetic perturbation screens to guide the development of new therapeutic agents for MM.

## Introduction

Multiple myeloma (MM) is caused by the clonal expansion of plasma cells in the bone marrow^1^. While survival from MM has improved significantly over the last decade with the introduction of immunomodulatory drugs, proteasome inhibitors and combination therapies, MM essentially remains incurable and many patients die following relapse^2^.

Targeting tumour-specific essential genes, which constitute a druggable dependency is being avidly pursued as a strategy for developing new therapeutic agents to treat cancer and overcome drug resistance. Our limited knowledge of cellular dependencies remains a barrier to this approach. To address this deficiency, the DepMap project has set out to exhaustively define the cellular dependencies operating in multiple cancer types using large-scale genetic perturbation platforms^3,4^.

**Figure 1:**
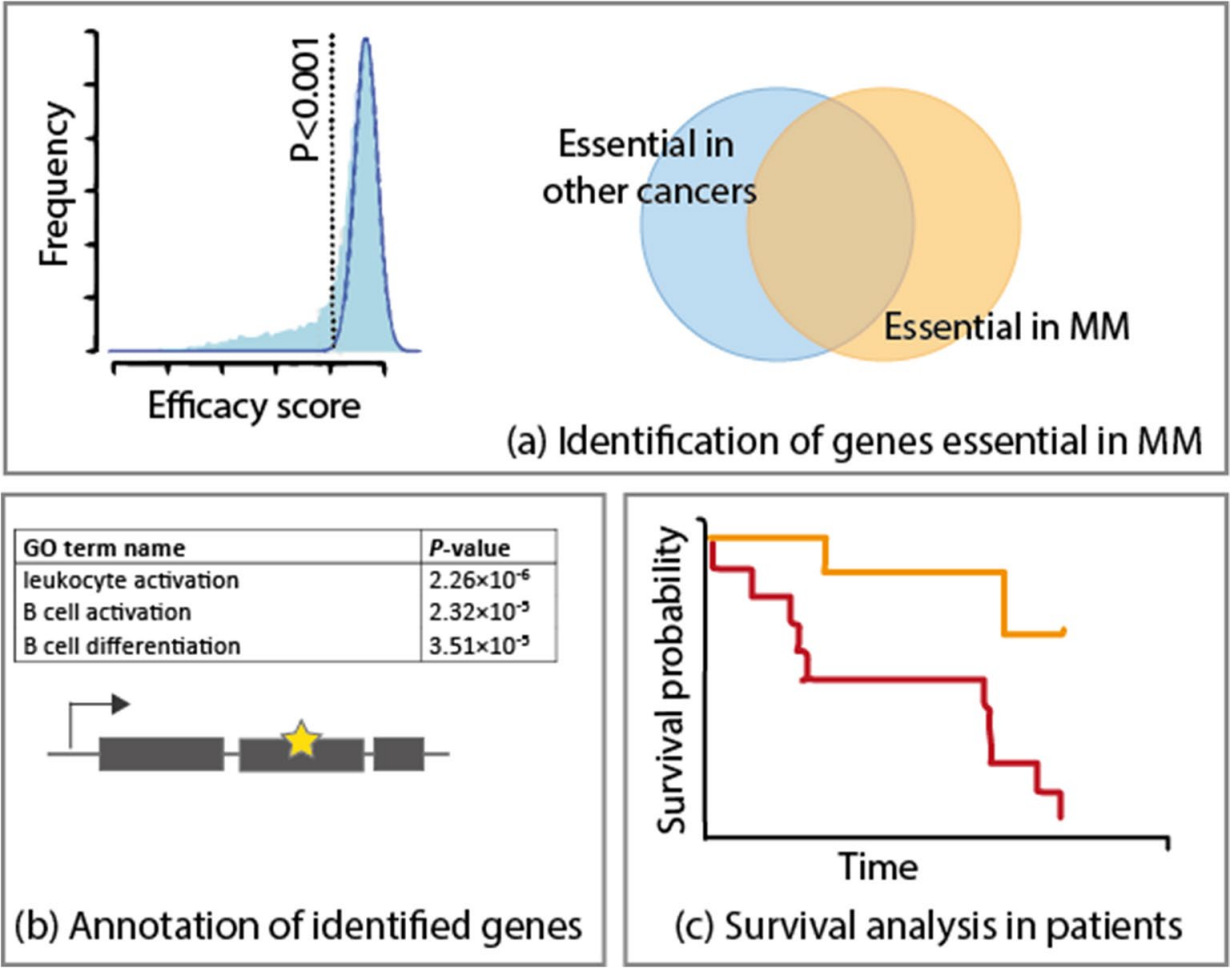
Overview of study design. (**a**) Identification of MM essential genes, (**b**) annotation of essential genes and (**c**) correlation of gene expression with patient survival.

To identify new druggable targets in MM, we analysed DepMap project data to identify gene and pathway dependencies and investigated if their expression influenced patient outcome (Fig. 1).

## Methods

### Data sources

The following data were obtained from DepMap 20Q2: cell line CRISPR scores (n = 769), cell line shRNA (n = 712), gene expression (n = 1305), and the cell line metadata (n = 1804) (ref.^4^). To study the relationship between gene expression and patient outcome we analysed data from three independent series of newly diagnosed MM: (1) RNA-seq and survival data on 797 patients in the Multiple Myeloma Research Foundation (MMRF) CoMMPass study (ref.^5^); (2) microarray (Affymetrix Human Genome U133Plus2.0) and survival data from 559 patients in the Total Therapy 2 (TT2) and Total Therapy 3 (TT3) trials^6,7^ (GEO accession GSE24080); and (3) microarray (Affymetrix HuGene 1.0 ST) and survival data on 147 patients (ArrayExpress E-MTAB-4032). Since all the data analysed was in the public domain, ethical approval for this study was not required. All studies from which data was obtained were conducted in accordance with the Declaration of Helsinki.

### Computation of perturbation scores

We adapted the methodology of Shimada et al.^8^ to compute unified perturbation scores based on DepMap shRNA and CRISPR efficacy metrics for all cell lines. We downloaded the preprocessed scores, which represent the growth effects of knocking the gene down or out, for shRNA and CRISPR respectively, from DepMap. In both datasets, a negative value in a particular cell line is indicative of its essentiality. In cases where there were missing values in either CRISPR or shRNA datasets, these data were imputed using a local polynomial regression (loess). After recovery of missing values, the unified perturbation score was computed as the weighted average of CRISPR and shRNA scores, such that S^θ^ = θS^C^ + (1 − θ)S^R^, where θ is the mixing ratio of the two scores, S^R^ is the shRNA score and S^C^ is the CRISPR score. We calculated the perturbation scores based on a range of different mixing ratios, with values of θ between 0 and 1 (Supplementary Fig. 1). Principal component analysis on these scores was performed and the first principal component between S^C^ and S^R^ was parallel to the line with θ = 0.68, which maximises the variance of (S^C^ S^R^). As a weighted average of CRISPR: shRNA = 60:40, is most similar to this principal component line, we chose θ = 0.6 for the rest of the analysis. This value also enabled the results to be consistent with the analysis of Shimada et al.^8^. In total 483 cell lines from DepMap had both shRNA and CRISPR data scores available for 15,765 genes.

### Definition of essential genes

Efficacy, a measure that indicates how essential a gene is in sensitive cell lines, was computed in the 25th percentile of MM cell lines. Here, this corresponded to eight out of 34 MM cell lines. While Shimada et al.^8^ used 1st percentile of all cancer cell lines for the majority of their analysis, given the small sample size of MM data available, we used the 25th percentile. Using a kernel density estimate function, a Gaussian curve was fit from the perturbation score distribution of the 25th percentile (X = 25) of MM cell lines. A gene was defined as essential when the score S^θ^_G,L(X=25)_ is lower than the essentiality threshold T_θ_, which is defined such that P(S^θ^_G,L(X=25)_ < T_θ_) = 0.001, where S^θ^_G,L_ ~ N(μ,σ) with μ, mean and σ, standard deviation. Using the same procedure, 449 non-MM cell lines were analysed to identify genes essential to other cell lines but not MM, using the 1st percentile (X = 1) of cell lines, which corresponded to 4 cell lines.

### Definition of selectivity

Selectivity is a measure of the cell line dependence of the response to the loss of a gene, with a more selective gene expected to have a greater varying effect across the population of cell lines. According to Shimada et al.^8^, this can be demonstrated through a greater dispersion of the score distribution for a selectively essential gene versus that for a commonly essential gene. Shimada et al.defined the dispersion of a gene as the difference between the 1-Xth and Xth percentile of S^θ^_G._ They found that the efficacy was linearly related for the majority of genes (commonly essential/non-essential) but that some genes had large positive residuals; these corresponded to the selectively essential genes. Shimada et al. ^8^ defined the selectivity U_θG,X_ using the residuals of the (100-X)-th percentile values for the efficacy:

$${\text{U}}_{{{\theta G},{\text{X}}}} = \frac{{{\text{R}}_{{{\theta G},{\text{X}}}} }}{{\widehat{{{\text{D}}_{{{\theta G},{\text{X}}}} }}}} = \frac{{{\text{E}}_{{{\theta G},100 - {\text{X}}}} - \widehat{{{\text{E}}_{{{\theta G},100 - {\text{X}}}} }}}}{{\widehat{{{\text{D}}_{{{\theta G},{\text{X}}}} }}}} = { }\frac{{{\text{D}}_{{{\theta G},{\text{X}}}} - \widehat{{{\text{D}}_{{{\theta G},{\text{X}}}} }}}}{{{\text{D}}_{{{\theta G},{\text{X}}}} }}$$

### Gene annotation

We downloaded mutation data from DepMap to annotate MM essential genes for mutations across 34 MM cell lines^9^.

### Gene-set enrichment analysis

Gene set enrichment analysis (GSEA) was performed to examine for the over-representation of shortlisted genes across Gene Ontology (GO) project annotations. Enrichment of GO term annotations obtained from GO.db^10^ were tested under a hypergeometric distribution.

### Relationship between gene expression and patient outcome

For each shortlisted gene, we compared MM-specific overall survival (OS) and progression-free survival (PFS) associated with low (bottom 25%) expression in the three patient series. For microarray data, CEL files were downloaded and processed with the oligo package in R using robust multichip average (RMA) normalisation in order to provide consistency^11^. For the RNA-seq dataset (CoMMPass) and microarray datasets (GSE24080 and E-MTAB-4032), OS was calculated from the date of diagnosis to death, and PFS was defined by the date of diagnosis and the date of disease progression or any death. Kaplan–Meier survival curves were generated and the log-rank test was used to test for difference in outcome between high and low gene expression. Cox regression analysis was used to calculate hazard ratio (HR) and 95% confidence intervals (CIs) associated with low (bottom 25%) expression, adjusting for age at diagnosis, sex and MM International Staging System (ISS) status. Meta-analysis of HRs was performed under a random-effects model to account for heterogeneity between datasets using the meta package in R^12^. We calculated Cochran’s *Q* statistic to test for between study heterogeneity. The Benjamini and Hochberg (BH) procedure was implemented to control for a false discovery rate (FDR) of 0.05 (ref.^13^).

### Druggability of essential genes

Data on the status of drug-target combinations were obtained by interrogation of the Open Targets platform^14^, which curates data on known and potential drug targets across diseases. Drug-targets combinations were cross-referenced using ChEMBL for information on development stage^15^.

## Results

**Figure 2:**
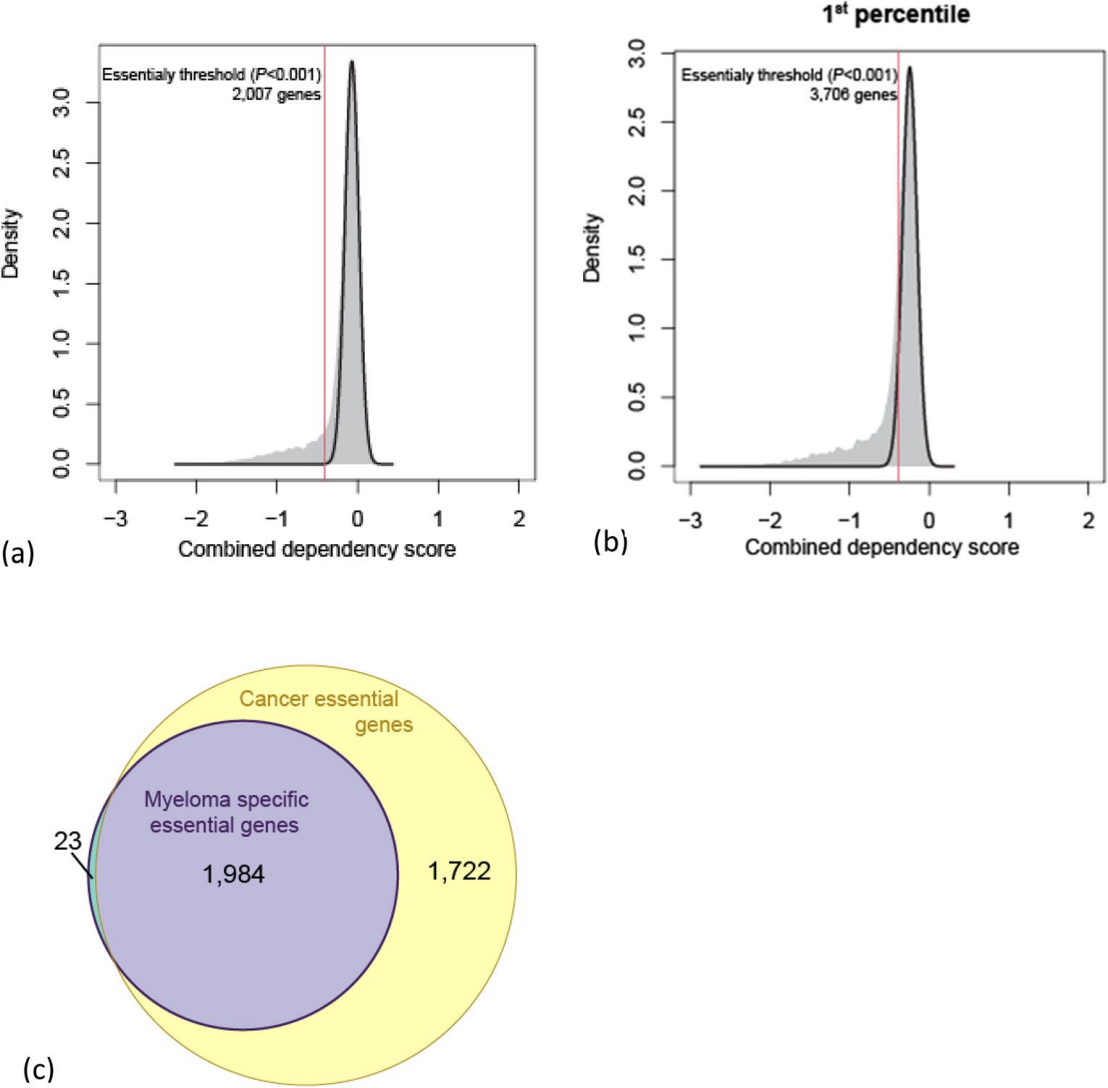
Identification of essential multiple myeloma genes. (**a**) Distribution of efficacy (perturbation scores of the 25th percentile most sensitive cell) among all the genes for MM cell lines. Fitted normal distribution is overlaid on distribution of genes. Red line shows efficacy score with *P* value < 1 × 10^−3^. (**b**) Distribution of efficacy (perturbation scores of the 1st percentile most sensitive) among all the genes for non-MM cell lines. Fitted normal distribution is overlaid on distribution of genes. Red line shows efficacy score with *P* value <  × 10^−3^. (**c**) Venn diagram demonstrating overlap of genes essential in MM and other cancer cell lines.

Unified perturbation scores were calculated, and in total 2007 genes (12.7%) were identified as being essential in MM and 3706 (23.5%) essential across all non-MM cancer cell lines. Of these, 1984 genes (12.5%) were common to both MM and all cancer cell lines, with 23 (0.15%) being essential only in MM (Fig. 2, Supplementary Tables 1a and b).

The most essential genes, as indicated by a more negative efficacy score, included both well-established genes ubiquitous in MM biology, including *NFKB1* and *PRDM1*, but also others such as *IRS1*. Genes with a high selectivity score included *MEF2C*, *NFKB1, RELB* and *IRS1. MEF2C* and *NFKB1* were both essential for MM and had high selectivity scores (Supplementary Table 1b).

To gain additional insight into how dysregulation of these essential genes might impact on MM biology, we performed GSEA and examined for mutations. Firstly, we performed GSEA to examine for over-representation of shortlisted genes among specific Gene Ontology (GO) annotations using the 23 genes identified as essential in MM. The GO terms spanned previously defined cancer hallmarks^16^ and signaling pathways involved MM, including NIK/NF-κB signaling, MAPK signaling, and B-cell proliferation. Notably, the most significantly overrepresented annotations were related to leukocyte activation, B-cell activation and B-cell differentiation (*P* = 2.26 × 10^−6^, *P* = 2.32 × 10^−5^ and *P* = 3.51 × 10^−5^, respectively; Supplementary Table 2). Furthermore, annotations related to cell proliferation, regulation of plasma cell differentiation and regulation of B-cell differentiation/activation were also significantly overrepresented. Using data from the Cancer Cell Line Encyclopaedia (CCLE) we tested for mutations in the 23 essential genes, across 34 MM cell lines and found 12 genes which harboured mutations in 17 cell lines (Supplementary Table 3).

**Figure 3:**
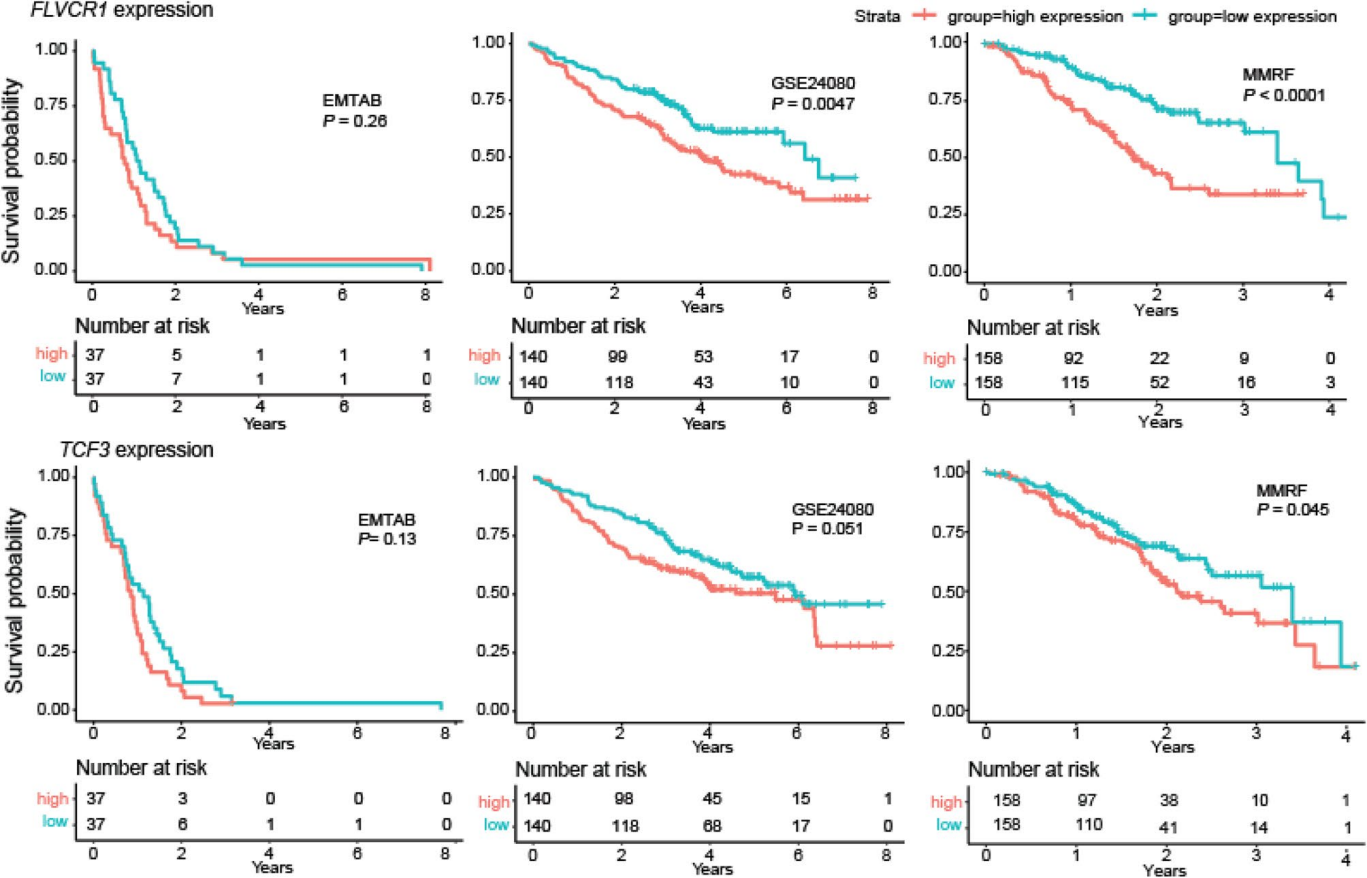
Kaplan–Meier curves for the relationship between expression of *FLVCR1* and *TCF3* and progression-free survival in three cohorts. The red line depicts the survival curve for higher (top 25%) expression of each gene, blue line depicts survival curve for lower (bottom 25%) expression of each gene. MMRF; MMRF CoMMpass IA10 RNA-seq. GSE20480 and E-MTAB-4032; microarray datasets.

Following on, we examined the relationship between expression of each of the 23 shortlisted genes with PFS and OS between patients with high (top 25%) and low (bottom 25%) tumour gene expression in each of the three datasets (Supplementary Table 4 and Supplementary Table 5). Low expression of *POU2AF1* was associated with worse OS (HR = 1.46, *P* = 0.025), while low expression of *AUP1* was associated with better OS (HR = 0.69, *P* = 0.028; Supplementary Figs. 2 and 3). Low expression of *FLVC1, TCF3, HERPUD1* and *POU2AF1* was significantly associated with PFS after correction multiple testing (*FLVCR1* HR = 0.57, *P* = 2.3 × 10^−4^; *TCF3* HR = 0.67, *P* = 7.6 × 10^−4^; *HERPUD1* HR = 1.42, *P* = 3.4 × 10^−3^; *POU2AF1* HR = 1.53*, P* = 6.8 × 10^−3^; Fig. 3 and Supplementary Figs. 4 and 5).

To investigate if any of the MM essential genes are currently, or are being considered as, drug targets in any cancer or non-malignant disease we queried the Open Targets^14^. Two of the 23 MM essential genes, *PIM2* and *IKBKB*, are catalogued as targets for six therapies across 19 disease categories: nine in the context of cancer including MM, non-Hodgkin lymphoma, and acute myeloid leukaemia (Supplementary Table 6).

## Discussion

By analysing DepMap project data we identified 23 gene-dependencies in MM. Essential genes were significantly overrepresented in annotations related to B-cell activation, including differentiation and the regulation of these pathways. Additionally, they were also enriched for ontologies related to cell proliferation, a hallmark of cancer^16^.

While interaction between genes is complex, if a gene is essential to survival of a cancer cell line, we may a priori expect a worse prognosis is likely to be associated with higher expression, as in the case of *FLVCR1* and *TCF3*.

A number of the genes our analysis highlights as potential drug targets have well established roles in MM, for example *MAF, IKZF3, NFKB1* (part of the NF-κB complex) and *PRDM1* **(**Supplementary Table 1). Notably, of the 1984 genes that were common to both MM and all cancer cell lines, 19 and 26 genes overlapped with those in the GEP70^17^ and SKY92^18^ gene prognostic risk scores, respectively (Supplementary Table 7).

Alongside more established MM genes, our analysis highlighted genes less studied in the context of MM, for example *FLVCR1* and *TCF3,* both of which were associated with MM survival (Fig. 3, Supplementary Table 4)*. TCF3,* has been shown to influence tumorigenicity and cell differentiation in breast cancer^19^. *FLVCR1* encodes Feline leukaemia virus subgroup C receptor 1, a membrane heme exporter protein. While the role of heme transport in MM remains to be established abnormal levels of heme have been reported to be essential for progression and metastasis in other tumour types^20,21^. For example, recent work has shown that silencing of *FLVCR1* led to inhibition of proliferation of synovial sarcoma cells in vitro and in vivo via regulation of cytotoxic autophagy^22^. Although speculative, since *FLVCR1* is located on 1q32, it raises the possibility that its expression may be responsible for the poor prognosis associated with 1q32 gain in MM^23–27^.

In addition, our analysis identified *IKBKB, CHUK and RELB* as essential in MM. These genes encode proteins which are play a role in NFκB signalling^28^, a pathway which is ubiquitous in MM. RNA interference screening has previously identified *CHUK* as a lenalidomide sensitiser in MM^29^. *CHUK* encodes a component of the IKK complex that plays a key role in NF-κB pathway activation^30^. *IKBKB* is currently undergoing investigation as a therapeutic target in pancreatic carcinoma, lymphoid neoplasms and melanoma (Supplementary Table 6). Furthermore, our analysis highlighted genes which have been linked to other cancers, including *SMAD7*, where overexpression of the gene is associated with worse prognosis in colorectal cancer^31^, and *PIM2*, which has been identified as an oncogene in multiple cancers including leukaemia, liver, lung, breast and prostate^32^. This gene has been shown to be involved in repressing the DNA-damage response in MM cells^33^. Another study found that *PIM2* promoted TSC2 suppression of mTOR-C1, via phosphorylation of TSC2 by PIM2, which drove MM cell proliferation^33^. *PIM2* is undergoing investigation as a therapeutic target in MM (Phase I trials) and three other cancers (Supplementary Table 6).

It is noteworthy that many of the genes we identified are not necessarily commonly somatically mutated/disrupted, emphasising the limitations of undue reliance on sequencing as a strategy for informing cancer drug development. Nonetheless, the identified genes have been linked to other cancers and have plausible roles in the development and progression of MM a priori.

A strength of our analysis is that that we have linked gene dependencies in cell line data to the relationship between gene expression and patient survival, seeking to prioritise candidates for novel therapeutic interventions. However, since we only had 80% power to demonstrate a HR of 1.46 and 1.30, for OS and PFS association with expression respectively, a failure to demonstrate a relationship for other essential genes may simply reflect study power if the effect is marginal (Supplementary Fig. 6). Ideally studying the relationship between gene expression and patient outcome is optimal within the context of a clinical trial where patients are treated in a uniform fashion and adjustment for any covariates can readily be undertaken. A limitation of our study is that our analyses are based on real world data from three publicly accessible cohorts and either no treatment information is provided, or patients have not been consistently treated. Excepting such limitations, it is the case that our findings are consistent across the three cohorts, supporting the robustness of our observations. The subtypes of MM which have chromosome t(4;14), t(11;14), t(14;16)/t(14;20) translocations or hyperdiploidy as an initiating event typically can have a different clinical course reflecting underlying biology^26,34–36^. Ideally it would be preferable to conduct analyses stratified by these driver lesions, however the number of cell lines for each MM subtype in DepMap is limited (Supplementary Table 8). Given that targeting of cereblon by IMiDs is highly effective across all MM, this deficiency does not necessarily invalidate our findings^37^. The use of cell lines for drug dependency studies can be questioned on the basis of how well they represent the behaviour of tumour cells in patients. However, long-term culture of MM plasma cells isolated from marrow-localized disease have so far been unsuccessful^38^. Furthermore, the effectiveness of drugs for treating cancer is invariably determined in part by normal tissue response. Here we have sought to prioritise genes as targets on the basis of selectivity using the non-MM cell lines as surrogates for corresponding normal tissues.

The high attrition rate of cancer drug development programs is a significant barrier to realising the promise of precision oncology^39^. Our analysis serves to illustrate that insights from large-scale genetic perturbation screens has the potential to guide drug development and repurposing to treat MM.

## Supplementary Information

Supplementary Information 1.

Supplementary Information 2.

## Data Availability

Cell line CRISPR scores, cell line shRNA, gene expression, and the cell line metadata were obtained from DepMap 20Q2 (https://depmap.org/portal/download/all/). RNA-seq and survival data on 797 patients in the Multiple Myeloma Research Foundation (MMRF) CoMMPass study were obtained from MMRF (https://themmrf.org/). Microarray (Affymetrix Human Genome U133Plus2.0) and survival data from 559 patients in the Total Therapy 2 (TT2) and Total Therapy 3 (TT3) trials were obtained from the Gene Expression Omnibus (GEO accession GSE24080). Microarray (Affymetrix HuGene 1.0 ST) and survival data on 147 patients were obtained from Array Express (E-MTAB-4032).
