# Supplementary material for: Exploiting gene dependency to inform drug development for multiple myeloma
Source: Sci Rep. 2022 Jul 26;12:12696. doi: 10.1038/s41598-022-16940-7 (PMC9325789; doi:10.1038/s41598-022-16940-7)
Supplement: Supplementary file 1 — Supplementary Information 1. [file 41598_2022_16940_MOESM1_ESM.docx]

**SUPPLEMENTARY INFORMATION**

**Exploiting gene dependency to inform drug development for multiple myeloma**

**Went *et al***

**
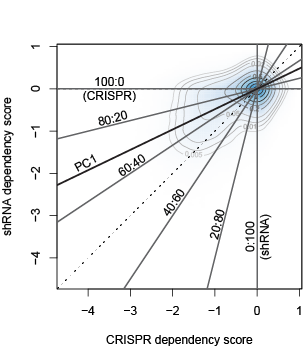
**

**Supplementary Figure 1: Principal component analysis of the CRISPR and shRNA efficacy datasets.** Dependency scores defined with different mixing ratios (θ) are computed by projecting each point onto the corresponding lines. PC1 is the direction of the primary principal component line. The unified perturbation score was computed as the weighted average of CRISPR and shRNA scores such that two such that S^θ^=θS^C^+(1−θ)S^R^, where θ denotes the fraction of CRISPR dependency scores, S^R^ is the shRNA score and S^C^ is the CRISPR score. The first principal component line (PC1) between S^C^ and S^R^ was parallel to the line with θ=0.68. Figure produced in R based on code from https://github.com/kenichi-shimada/depmap-analysis.


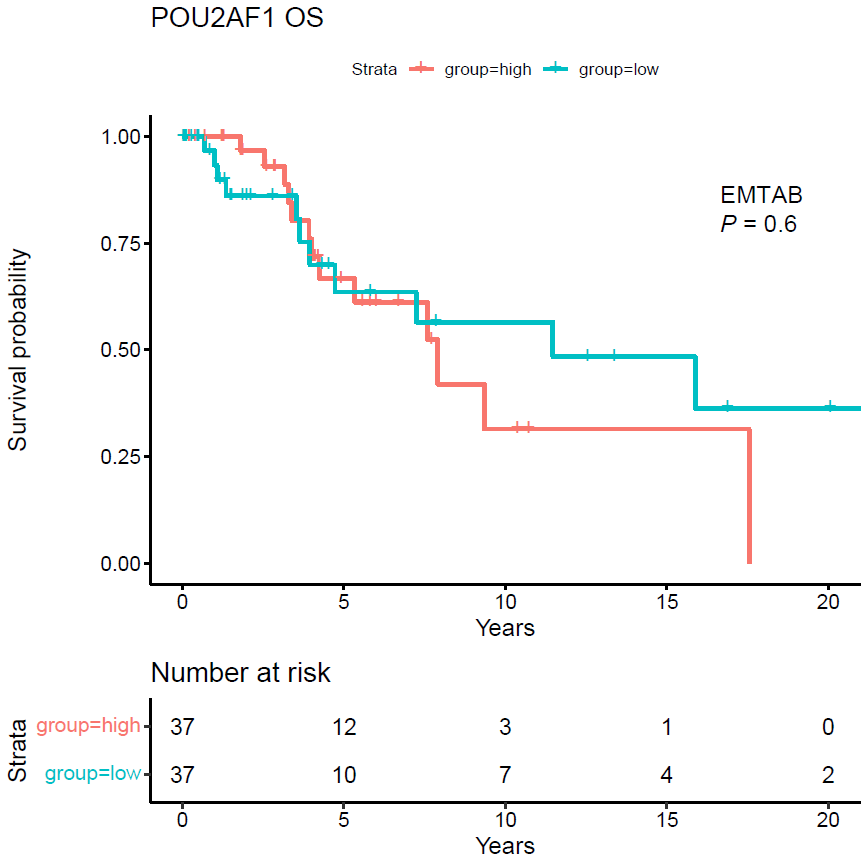

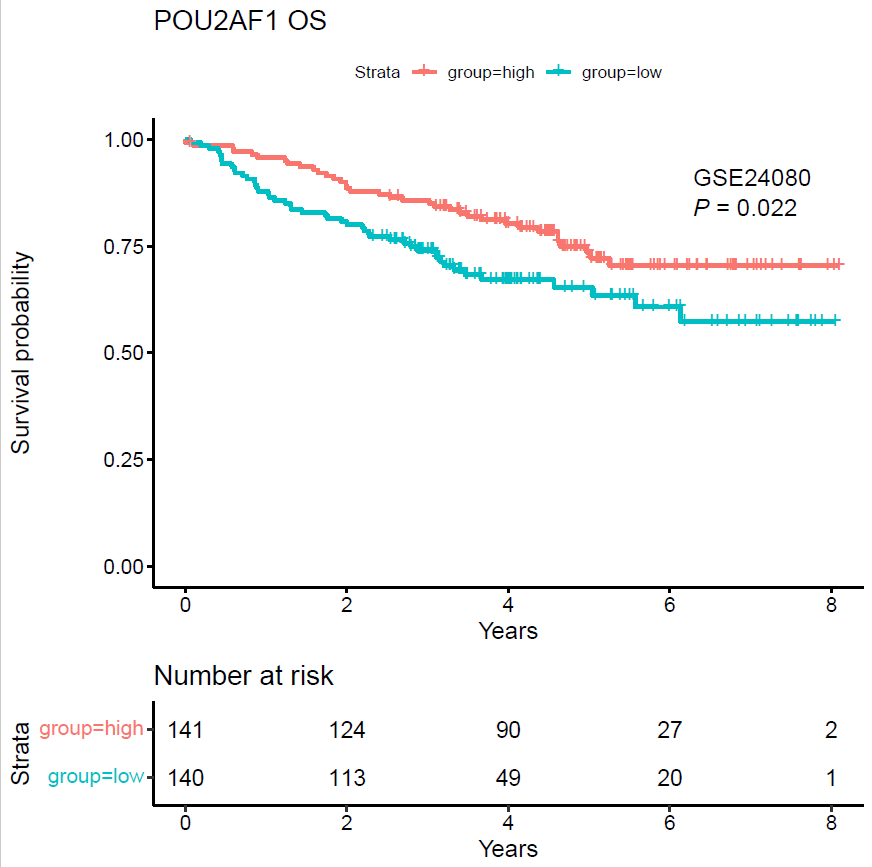

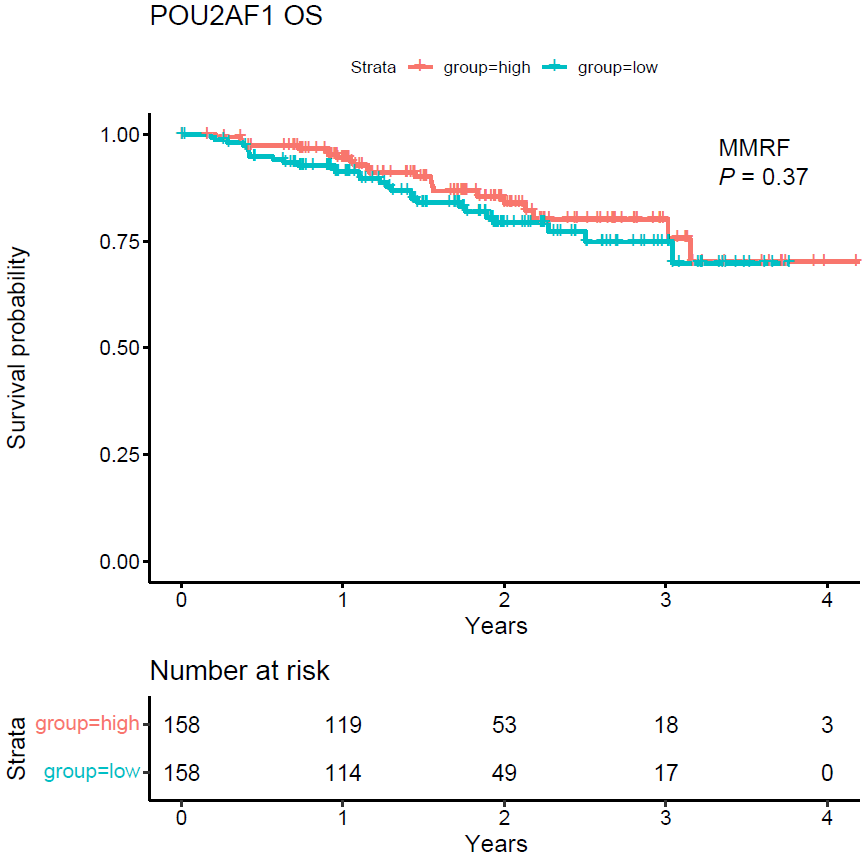


**Supplementary Figure 2: Kaplan-Meier curves showing the relationship between *POU2AF1* expression and overall survival (OS) in three cohorts.** Figures produced in R using survminer package.


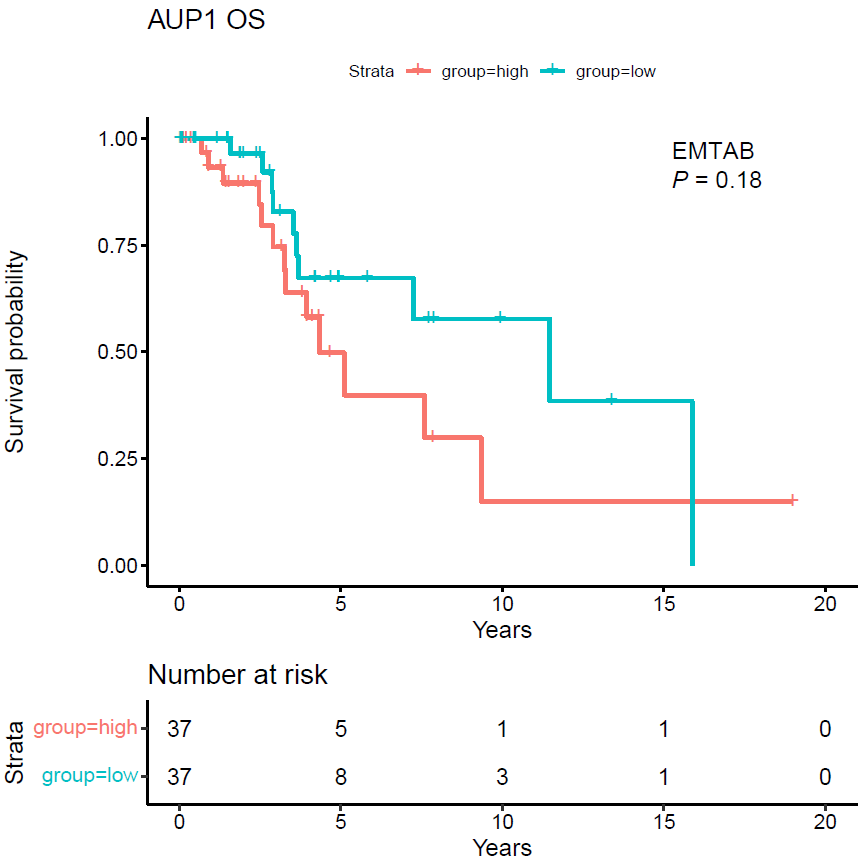

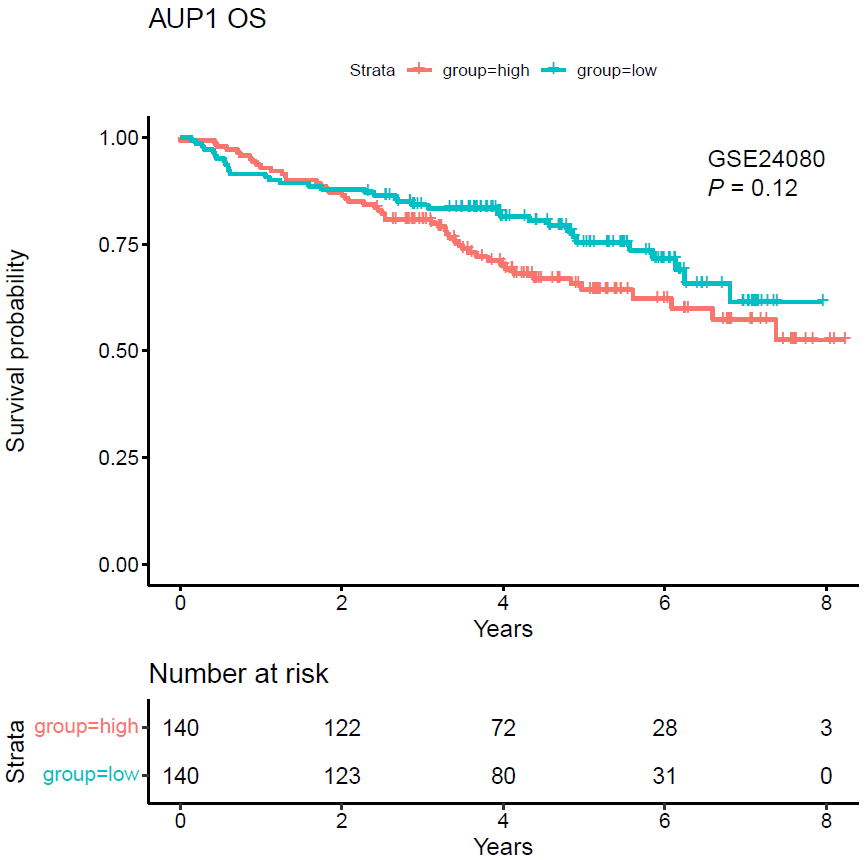

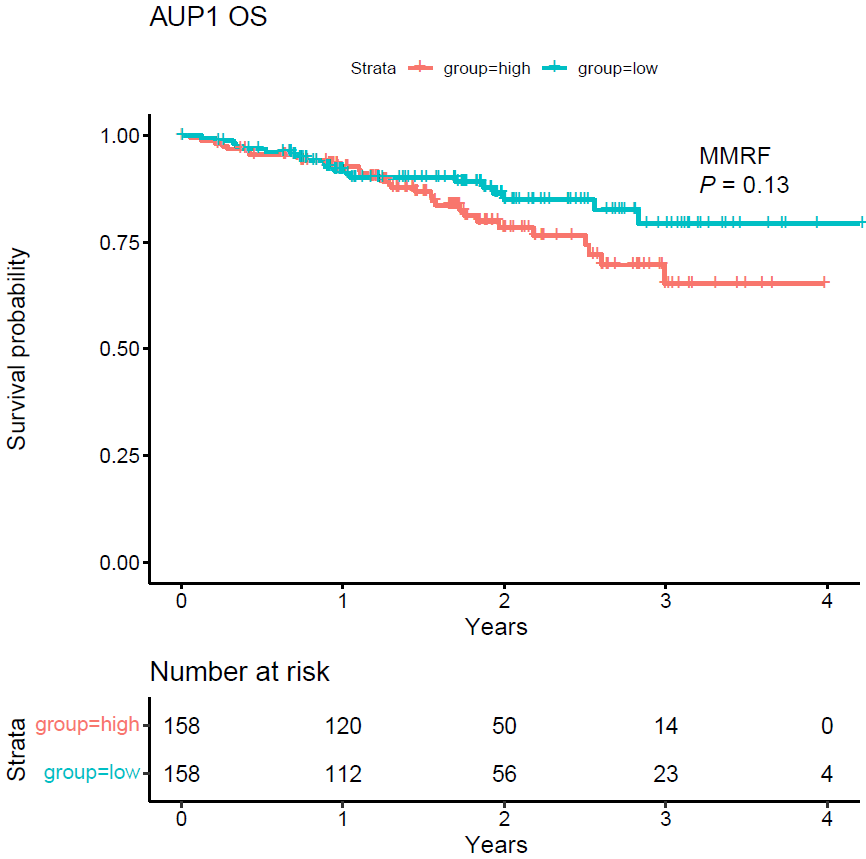


**Supplementary Figure 3: Kaplan-Meier curves showing the relationship between *AUP1* expression and overall survival (OS) in three cohorts.** Figures produced in R using survminer package.


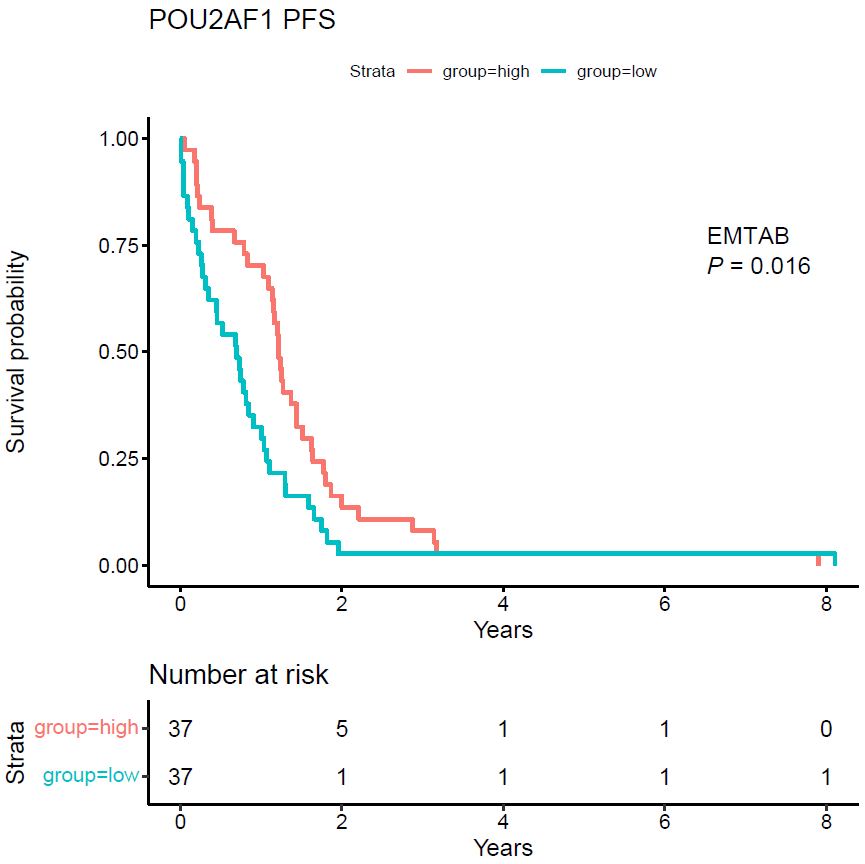

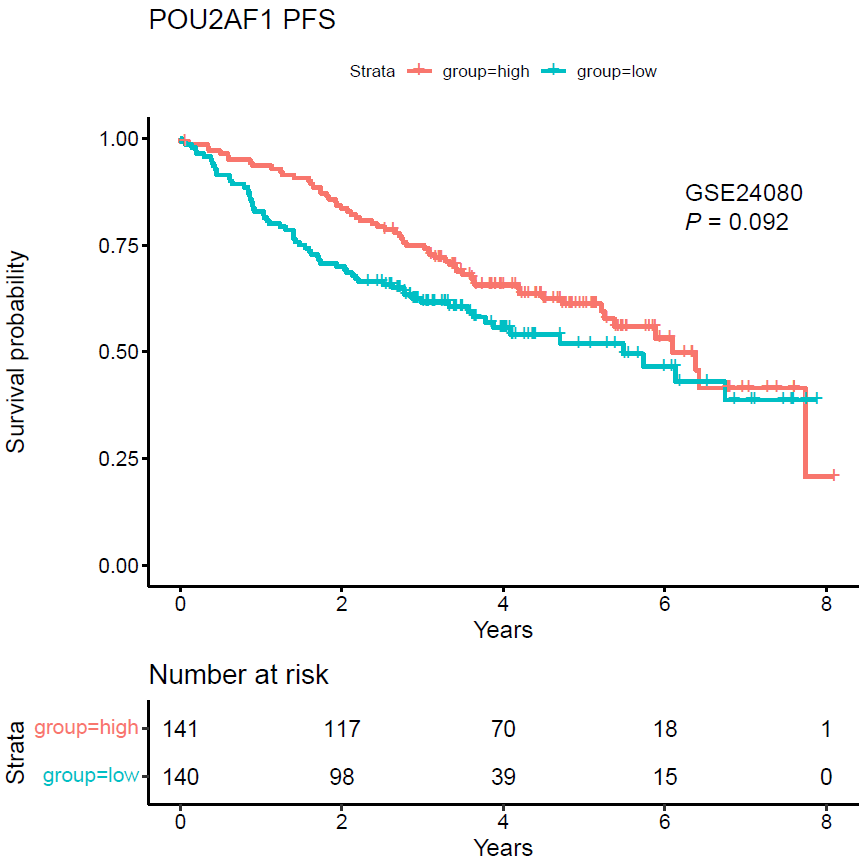

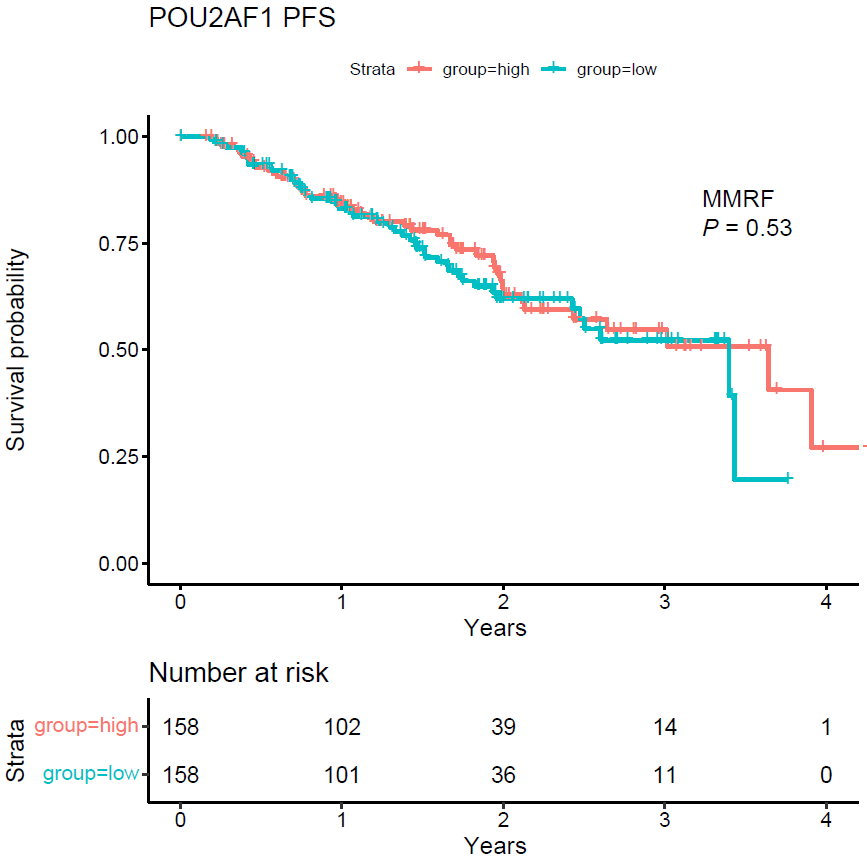


**Supplementary Figure 4: Kaplan-Meier curves showing the relationship between *POU2AF1* expression and progression-free survival (PFS) in three cohorts.** Figures produced in R using survminer package.


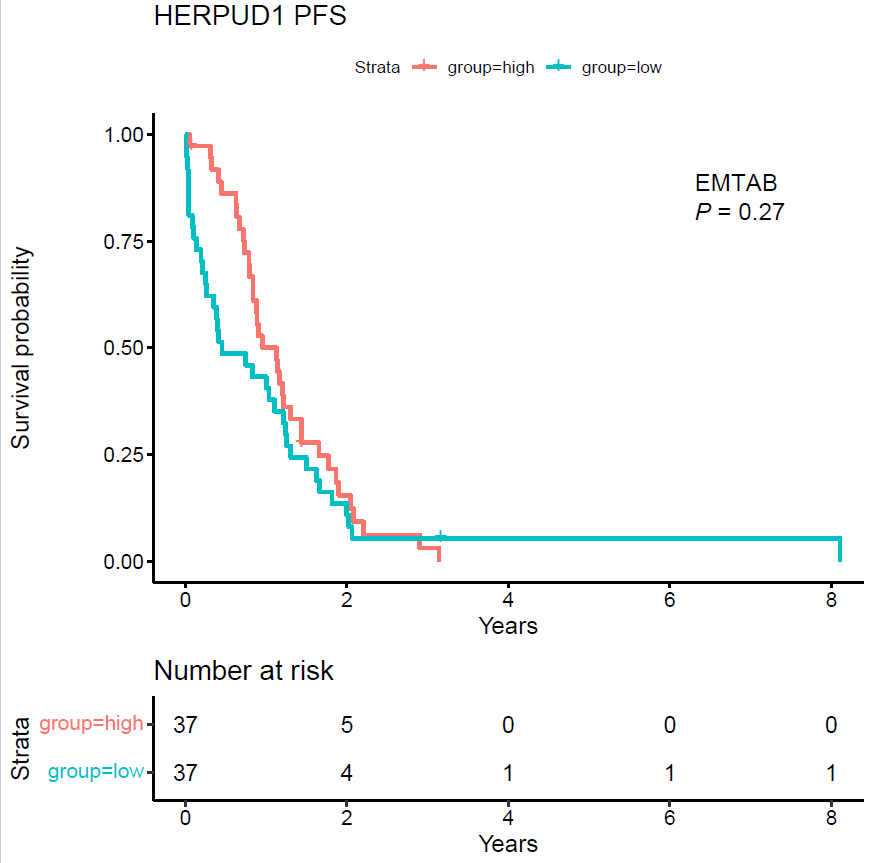

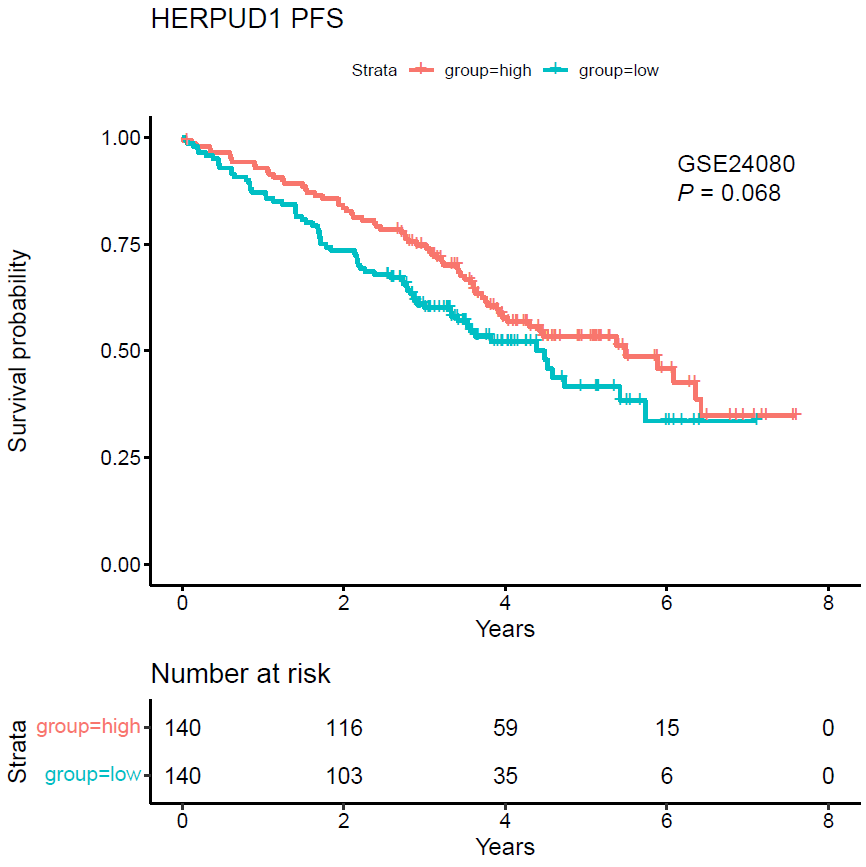

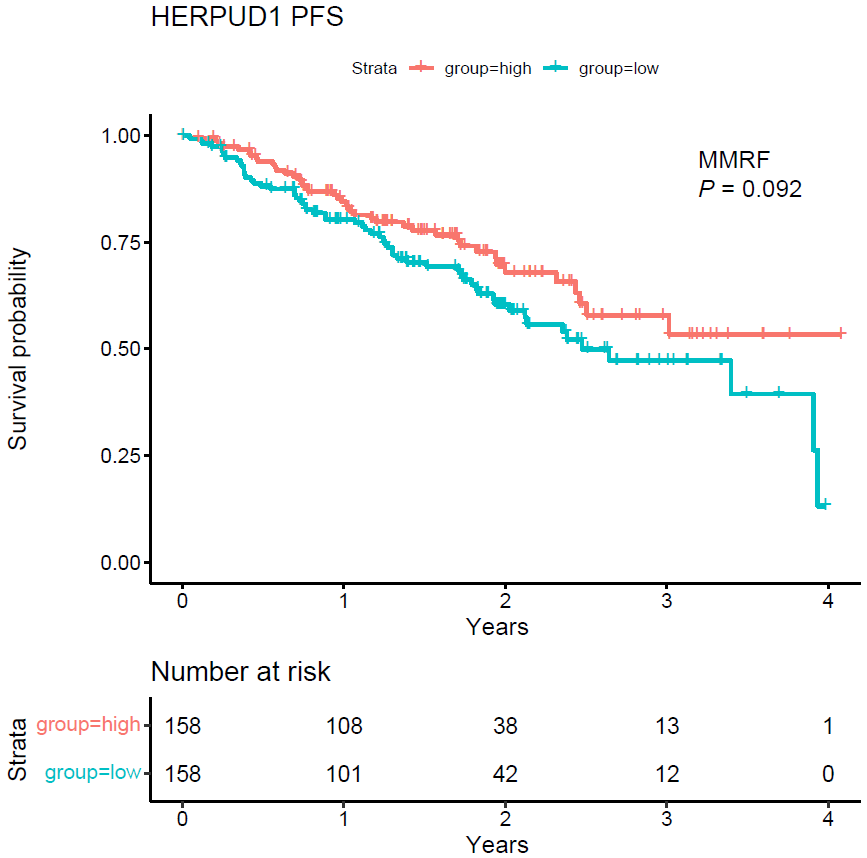


**Supplementary Figure 5: Kaplan-Meier curves showing the relationship between *HERPUD1* expression and progression-free survival (PFS) in three cohorts.** Figures produced in R using survminer package.


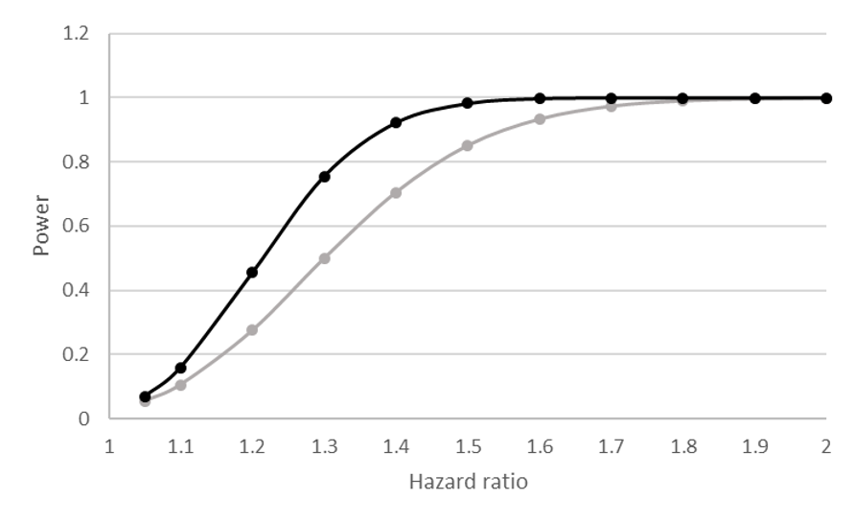


**Supplementary Figure 6: Power of survival analysis across a range of hazard ratios.** Power was calculated using powerSurvEpi package in R, based on n=375 individuals in both the lower and higher expression groups. An overall survival (OS) probability of 0.3 and progression-free survival (PFS) probability of 0.55 was used, which was in line with the clinical characteristics of samples. Grey line represents OS and black line represents PFS.
